# The Conformation of the N-Terminal Tails of *Deinococcus grandis* Dps Is Modulated by the Ionic Strength

**DOI:** 10.3390/ijms23094871

**Published:** 2022-04-28

**Authors:** João P. L. Guerra, Clement E. Blanchet, Bruno J. C. Vieira, Ana V. Almeida, João C. Waerenborgh, Nykola C. Jones, Søren V. Hoffmann, Pedro Tavares, Alice S. Pereira

**Affiliations:** 1 Associate Laboratory i4HB, Institute for Health and Bioeconomy, NOVA School of Science and Technology, Universidade NOVA de Lisboa, 2829-516 Caparica, Portugal; jp.guerra@campus.fct.unl.pt (J.P.L.G.); acv.almeida@campus.fct.unl.pt (A.V.A.); 2 UCIBIO—Applied Molecular Biosciences Unit, Department of Chemistry, NOVA School of Science and Technology, Universidade NOVA de Lisboa, 2829-516 Caparica, Portugal; 3 European Molecular Biology Laboratory, Hamburg Outstation, Notkestrasse 85, 22603 Hamburg, Germany; clement.blanchet@embl-hamburg.de; 4 Centro de Ciências e Tecnologias Nucleares, DECN, Instituto Superior Técnico, Universidade de Lisboa, Estrada Nacional 10, 2695-066 Bobadela, Portugal; brunovieira@ctn.tecnico.ulisboa.pt (B.J.C.V.); jcarlos@ctn.tecnico.ulisboa.pt (J.C.W.); 5 ISA, Department of Physics and Astronomy, Aarhus University, DK-8000 Aarhus C, Denmark; nykj@phys.au.dk (N.C.J.); vronning@phys.au.dk (S.V.H.)

**Keywords:** DNA-binding protein from starved cells (Dps), *Deinococcus grandis*, N-terminal tail extensions, mini-ferritins, biological small-angle X-ray scattering, conformational changes, Mössbauer spectroscopy

## Abstract

DNA-binding proteins from starved cells (Dps) are homododecameric nanocages, with N- and C-terminal tail extensions of variable length and amino acid composition. They accumulate iron in the form of a ferrihydrite mineral core and are capable of binding to and compacting DNA, forming low- and high-order condensates. This dual activity is designed to protect DNA from oxidative stress, resulting from Fenton chemistry or radiation exposure. In most Dps proteins, the DNA-binding properties stem from the N-terminal tail extensions. We explored the structural characteristics of a Dps from *Deinococcus grandis* that exhibits an atypically long N-terminal tail composed of 52 residues and probed the impact of the ionic strength on protein conformation using size exclusion chromatography, dynamic light scattering, synchrotron radiation circular dichroism and small-angle X-ray scattering. A novel high-spin ferrous iron-binding site was identified in the N-terminal tails, using Mössbauer spectroscopy. Our data reveals that the N-terminal tails are structurally dynamic and alter between compact and extended conformations, depending on the ionic strength of the buffer. This prompts the search for other physiologically relevant modulators of tail conformation and hints that the DNA-binding properties of Dps proteins may be affected by external factors.

## 1. Introduction

Mini-ferritins, a subfamily of the ferritin family of proteins, play a major role in the prokaryotic defense mechanism against DNA damage, to the extent that the term is almost interchangeable with Dps proteins (DNA-binding proteins from starved cells) [1,2]. DNA protection by Dps proteins is achieved through two distinct, but interconnected, activities, as follows: DNA binding and condensation (direct physical shielding) [3], and through their ferroxidation and iron mineralization activity (indirect chemical protection) [4], which decreases oxidative stress by preventing the production of reactive oxygen species via Fenton chemistry. Thus, the expression of *dps* genes occurs mostly at the starvation-induced stationary phase of bacterial growth, when it becomes the most abundant protein in the nucleoid, or during acute oxidative stress caused by toxic levels of hydrogen peroxide [5,6].

The structure–function relationship of Dps proteins has been the subject of extensive research since their discovery, with homologues from over 50 different organisms currently characterized in the literature and several hundred encoding genes annotated in genomic databases [2]. These proteins exhibit a striking degree of conservation, with virtually all structures currently deposited in the PDB, displaying a 9–10 nm wide hollow cuboid with rounded corners and P23 symmetry [7]. This structure results from the assembly of 12 identical subunits, composed of a conserved four-helix bundle. The iron-binding sites (ferroxidase centers), where the Fe^2+^ ions are oxidized by hydrogen peroxide [4] or molecular oxygen [8], are located at each dimer interface and are formed by amino acid residues of adjacent subunits. The four-helix bundle is flanked by N- and C-terminal extensions of variable length and amino acid composition. These extensions are generally unstructured and flexible, as most crystal structures are unable to completely model their position.

The N- and C-terminal tails have recently gathered interest in Dps research. Believed to be responsible for providing the interface needed for the dodecamer assembly, they have also been linked to the DNA binding activity [3,9,10]. The importance of the N-terminal tail of several Dps homologues has been described, particularly of the *Escherichia (E.) coli* Dps [3,11,12], *Mycobacterium (M.) smegmatis* Dps1 [13,14] and Dps2 [15], *Lactococcus lactis* DpsA and DpsB [16], *Marinobacter (M.) hydrocarbonoclasticus* Dps [17] or *Deinococcus (D.) radiodurans* Dps1 and Dps2 [18,19,20] proteins. The presence of several positively charged amino acid residues is a common feature at the distal end of the tail, with Dps homologues that have a high affinity for DNA binding usually possessing three to seven lysine residues. These residues are crucial for establishing protein–DNA interactions since their deletion or substitution impairs the formation of DNA-Dps complexes [11]. Nevertheless, the overall electrostatic potential of the tail is also of importance, as a single charge modification in the N-terminus of *M. hydrocarbonoclasticus* Dps renders it unable to bind to DNA [17]. In some cases, such as the *D. radiodurans* Dps1, the proximal end of the N-terminal tail (at the outer surface of the protein) also presents an extra metal-binding site that may be occupied by divalent metals, such as Zn^2+^, Co^2+^ or Mn^2+^, thought to be important for dodecamer assembly and DNA binding [19,21,22].

The shape and orientation and solvent accessibility of the longer N-terminal tails is of particular interest and believed to be related to their function. The spatial constraints of the crystallization process force the N-terminal tails to adhere to the dodecamer shell in every crystal structure solved thus far, with the protein presenting a compact globular shape. However, small-angle X-ray scattering (SAXS) data in solution revealed that the N-terminal tails of *D. radiodurans* Dps1 and Dps2 were protruding outwards from the dodecameric cage, in a symmetric star-like conformation. In this conformation, the amino acid residues of the tails are exposed to the solvent and accessible for DNA binding [23]. The model generated for an artificial tailless version of the protein showed a dodecamer sphere with no protrusions, in accordance with the crystallographic structure of the protein. Recent SAXS data of *E. coli* Dps-DNA co-crystals revealed that the buffer composition and the presence of divalent metals, such as Mg^2+^ or Fe^2+^, can affect the conformation of the protein and disrupt the Dps–DNA interactions [24,25].

In this work, we further explore the structural dynamics of the Dps N-terminal tails by using dynamic light scattering (DLS), synchrotron radiation circular dichroism (SRCD) and small-angle X-ray scattering (SAXS) to probe the effect of the ionic strength of the buffer on the conformation of *Deinococcus grandis* Dps, a hitherto uncharacterized homologue of *D. radiodurans* Dps1 with a N-terminal tail over 50 residues long. Furthermore, we characterize the Mössbauer parameters of a novel ferrous iron-binding site within the tail and discuss its possible relevance for protein function.

## 2. Results

### 2.1. Expression and Characterization of Proteins

The protein sequence WP_058978256.1 (NCBI protein database) was identified as a Dps homologue, by querying several known Dps sequences against the genome of *Deinococcus grandis* using Protein BLAST (here abbreviated as DgrDps). The sequence exhibits 77% identity (EMBOSS Needle [26]) with its close relative *D. radiodurans* Dps1, 23% with the *E. coli* Dps and 20% with the *M. smegmatis* Dps1, three of the Dps homologues best characterized so far. This transcript codes for a protein subunit with 205 amino acid residues (22536 Da, pI = 5.41), predicted (by PSIPRED [27] and SWISS-MODEL [28]) to fold as the typical four-helix bundle found in all Dps proteins, with an unordered N-terminal tail comprising 52 residues (of which 9 are lysines), but no significant C-terminal tail. A tailless variant of the protein, lacking the initial 46 residues (abbreviated as DgrDps ∆N) was also engineered (18042 Da subunit, pI = 4.61). Both the wild type (WT) and ∆N variant of DgrDps were overexpressed in *E. coli* using standard molecular biology techniques.

**Figure 1:**
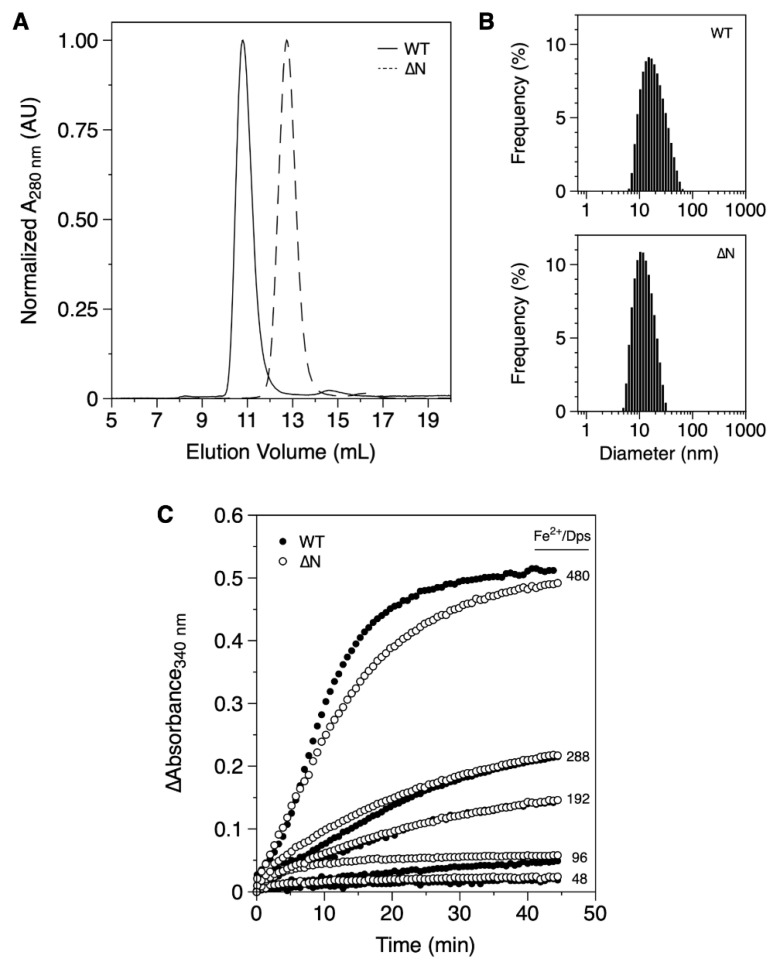
Biochemical characterization of purified fractions of DgrDps WT and ∆N variant. (**A**) SEC elution profiles of DgrDps WT (full line) and ∆N (dashed line); (**B**) DLS particle size distribution of DgrDps WT and ∆N variant samples; (**C**) progress curves of the Fe^2+^ ions oxidation reaction in the presence of O_2_, for different amounts of the iron substrate (Fe^2+^/protein molar ratios of 48, 96, 192, 288 and 480). In all experiments the protein was buffered in 200 mM MOPS pH 7.0 and 200 mM NaCl.

The recombinant expression of DgrDps WT yielded the protein in the insoluble fraction and required solubilization with urea, while DgrDps ∆N was produced in the cytosol, even though expression levels were similar. Ultimately, at the final stage of purification, both protein fractions were pure and monodisperse (Figure 1A,B).

The apparent molecular masses of both WT and ∆N proteins were determined by SEC. The SEC profile of DgrDps WT in 200 mM MOPS pH 7.0 and 200 mM NaCl (Figure 1A) shows a single sharp peak at ~11 mL, which, according to the calibration curve of the column, corresponds to an apparent molecular mass of 405 kDa, considerably higher than the prediction of a dodecamer with 270.2 kDa (deduced from the amino acid sequence translated from the encoding gene). The hydrodynamic diameter was estimated using DLS (Figure 1B). The Z-average value was 15.3 ± 0.2 nm, also above the value determined from the crystal structures of the Dps proteins (~10 nm considering the solvation sphere). This discrepancy can probably be explained by the positioning of the N-terminal tails, which were likely to be in the extended, star-shaped conformation as described for the *D. radiodurans* Dps1, which resulted in a SAXS envelope, ~15 nm wide [23].

The SEC and DLS data (Figure 1A,B) of the DgrDps ∆N variant, lacking the initial 46 amino acid residues, are consistent with a protein of smaller dimensions, presenting a chromatographic peak at ~13 mL (equivalent to an apparent molecular mass of 160 kDa) and a Z-average value of 10.8 ± 0.7 nm, corresponding to a tailless Dps dodecamer, as expected.

The iron ferroxidation and mineralization properties of both proteins were assessed by monitoring the absorbance at 340 nm, after the addition of different amounts of iron substrate (Fe^2+^/Dps molar ratios ranging from 48 to 480) in oxygen saturation conditions (Figure 1C). In this assay, ferrous ions are oxidized by molecular oxygen, one of the known co-substrates of Dps, forming ferric species that absorb between 300 and 400 nm. Once oxidized, the ferric species are translocated to the inner cavity, where nucleation and mineral (ferrihydrite) growth occur. For both proteins (DgrDps WT and ∆N), the progress curves are similar, which suggests that the artificial removal of the N-terminal tail did not drastically alter the functional and structural properties of the Dps dodecamer.

### 2.2. Conformational Dynamics

**Figure 2:**
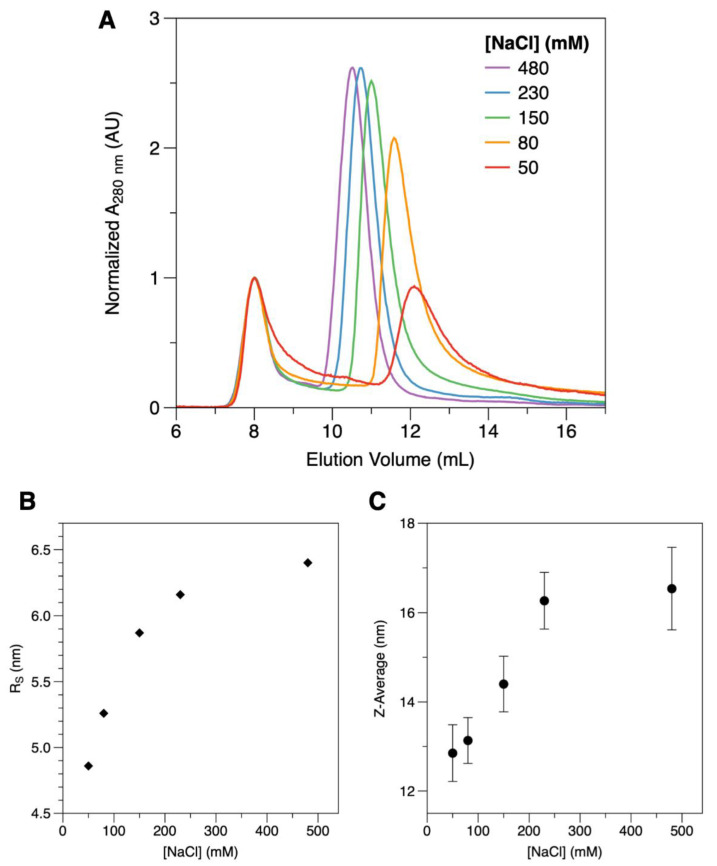
Impact of the ionic strength on the structure of DgrDps WT. (**A**) SEC elution profiles of DgrDps WT in 50 mM MOPS pH 7.0 buffer containing varying concentrations of NaCl, between 480 mM (purple), 230 mM (blue), 150 mM (green), 80 mM (orange) and 50 mM (red), co-eluted with blue dextran for determination of the void volume. (**B**) Ionic strength dependence of the Stokes radius (R_S_) estimated by SEC; (**C**) Hydrodynamic diameter (Z-average) variation with buffer salt concentration, as determined by DLS.

Even though the final product of the DgrDps purification is stable and homogeneous in the storage buffer (200 mM MOPS pH 7.0, 200 mM NaCl), DgrDps WT fractions presented two instances of somewhat abnormal behavior, when compared to the following information already known for other Dps proteins: the formation of inclusion bodies during overexpression and subsequent need for solubilization with urea (also observed for *D. radiodurans* Dps1 [21]), and increased propensity to aggregate during dilution or concentration steps. The fact that this behavior was not observed with DgrDps ∆N led us to hypothesize that the N-terminal tails of DgrDps WT could play an important role on protein stability, possibly associated with solvent accessibility, length, and the unstructured nature of the N-terminal tails. To probe the impact of the ionic strength of the buffer on the stability of the protein, protein samples were prepared in 50 mM MOPS, pH 7.0, containing varying concentrations of sodium chloride (from 50 to 480 mM). The resulting protein samples were analyzed by SEC. The chromatograms, normalized to the blue dextran peak at 8 mL, denote two distinct (but probably correlated) effects (Figure 2A). First, the amount of protein lost to aggregation in the centrifugation step, performed after the buffer exchange and before the injection, depends on the ionic strength of the buffer. Although the amount of protein per sample is the same before exchanging the buffer, the global area of the peak decreases with lower buffer ionic strengths. Secondly, the elution volume of the protein band increases with the ionic strength of the buffer (shifting from 10.5 mL at 480 mM NaCl, purple line, to 12.2 mL at 50 mM NaCl, red line), indicative of changes in the overall shape (the Stokes radius) of the protein. The estimated Stokes radius of the protein (Figure 2B) decreases from around 6.4 nm to 4.9 nm, as the salt concentration decreases from 480 mM to 50 mM. This shift was consistent with the particle size assessment by DLS (Figure 2C). In this case, the hydrodynamic diameter (Z-average) of the protein particle was calculated to be of 16.5 ± 0.9 nm at 480 mM NaCl and to gradually shift to 12.9 ± 0.6 nm at 50 mM NaCl. The polydispersity index (PI) remained at values below 0.3 throughout the experiment, expressing a high degree of protein homogeneity [29], regardless of the buffer condition tested.

**Figure 3:**
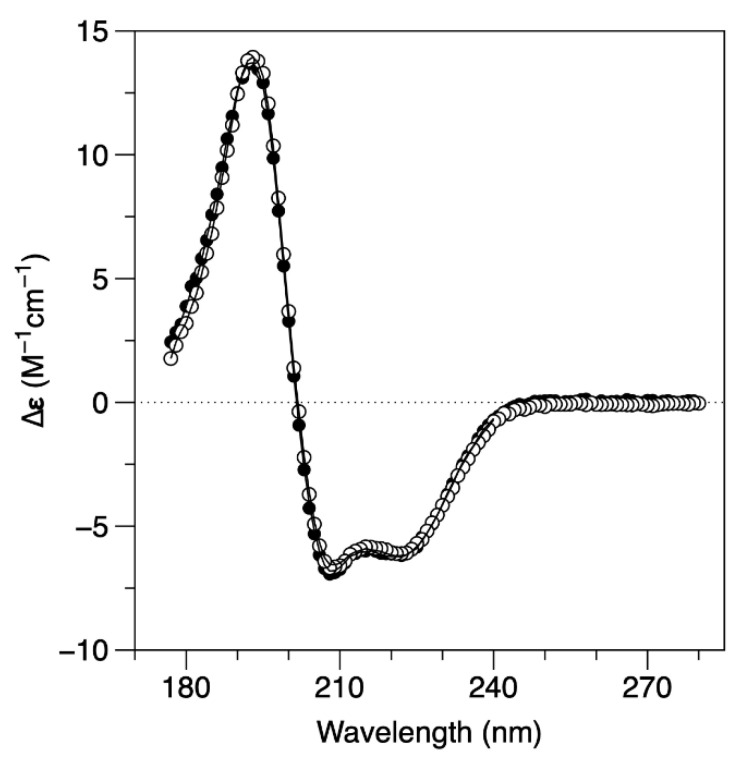
SRCD circular dichroism spectra of DgrDps WT in 10 mM MOPS pH 7.0 buffer, containing either 240 mM NaF (full circles) or 60 mM NaF (empty circles) at 25 °C. Solid line represents reconstructed spectral data from DichroWeb analysis.

The analysis of the secondary structure of the protein by SRCD (Figure 3) suggests that the structural rearrangement that occurs in this ionic strength dependent process happens without affecting the overall secondary structure of the protein. SRCD spectra were collected at the AU-CD beamline on the synchrotron radiation facility ASTRID2 at ISA, the Department of Physics and Astronomy, Aarhus University, Denmark. The analysis of the SRCD spectrum, obtained in 10 mM MOPS pH 7.0 and 240 mM NaF (full circles), is identical to the one obtained in 10 mM MOPS pH 7.0 and 60 mM NaF (empty circles). The secondary structure composition, estimated using DichroWeb, is 63−64% α-helix, less than 5% β-sheet, and 31−32% of unordered structures for both buffer conditions. These data are also in agreement with the expected secondary structure content of the DgrDps WT determined by homology modelling. A comparative analysis of the results from the SEC, DLS and SRCD data is displayed in Table 1.

Solution SAXS data were obtained for DgrDps WT and ∆N proteins samples in various ionic strength conditions, to better understand the conformational changes described above. Data were collected at the P12 beamline of EMBL on the PETRAIII storage ring (DESY, Hamburg, Germany) [30], using each corresponding buffer solution as an elution buffer for solvent reference.

**Figure 4:**
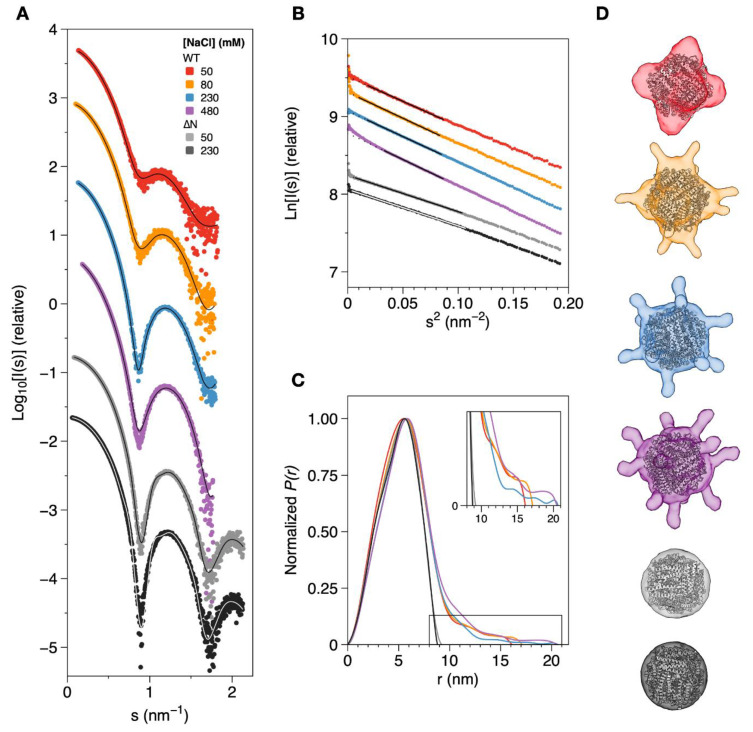
SAXS data and ab initio modelling of DgrDps WT and ∆N variant in different ionic strength conditions. (**A**) Experimental scattering curves and calculated fits (solid lines) of DgrDps samples in 50 mM MOPS pH 7.0 buffer, containing varying NaCl concentrations, which are as follows: 50 mM (red), 80 mM (orange), 230 mM (blue) and 480 mM (purple) for DgrDps WT and either 50 mM (light gray) or 230 mM NaCl (dark grey) for the DgrDps ∆N variant. (**B**) Guinier plots and linear fits (solid lines on top of the experimental points) of the scattering profiles shown in (**A**); (**C**) pair distance distribution curves; (**D**) representative ab initio models of DgrDps WT (generated by GASBOR) and ∆N (generated by DAMMIN) in the different sample conditions tested, superimposed with the dodecamer model for each protein (ribbons).

Overall, the scattering profiles of all the samples (Figure 4A) are similar and consistent with a particle shaped as a hollow sphere [31], presenting a good signal-to-noise ratio, at least until s = 2 nm^−1^. The Kratky plots (Figure S1 of the Supplementary Materials) display consecutive bell-shaped Gaussian curves of decreasing intensity, consistent with properly folded and homogeneous globular proteins. A Guinier analysis of the scattering data (Figure 4B) shows good linearity for all the samples and a reasonably small deviation at low s^2^ values (below 0.01 nm^−2^) for all samples, suggesting low levels of aggregation or radiation damage. The Guinier plots were used to estimate the radius of gyration (*R*_g_), which ranges from 4.38 nm to 4.67 nm for DgrDps WT as the ionic strength of the buffer increases. The *R*_g_ for the ∆N variant was calculated as 3.72 nm, regardless of the NaCl concentration (50 mM or 230 mM).

The representation of the pair distance distribution functions, *P*(*r*), calculated using GNOM [32] (Figure 4C), render sightly asymmetrical bell-shaped curves with a maximum at ~5.8 nm for all the buffer conditions and a sharp decay until 10 nm, which is characteristic of a spherical protein with a hollow core. The *D*_max_ for the tailless DgrDps ∆N variant is ~9.0 nm for both the ionic strength conditions tested, which is very close to the diameter of the dodecamer cage found in the crystal structures of Dps proteins [2]. On the other hand, the *D*_max_ of the WT protein samples reflect the presence of distended N-terminal tails. Instead of reaching *P*(*r*) = 0 at a diameter of approximately 10 nm, the distance distribution plot exhibits a second shape from *r* = 10 to 20 nm, depending on the ionic strength of the buffer. The *D*_max_ for the lowest NaCl concentration (50 mM, red curve in Figure 4C) was determined as 16.1 nm, whereas the highest *D*_max_ values were obtained for the samples at 230 mM (blue curve) and 480 mM NaCl (purple) at 20.6 and 20.5 nm, respectively. The *R*_g_ values, derived from *P*(*r*) calculation by GNOM, are consistent with the Guinier analysis and vary between 4.43 and 4.87 nm. A comprehensive comparison of the results and parameters calculated from the 1D dataset analysis is displayed in Table 2.

The ab initio modelling tools DAMMIN and GASBOR were used to determine the low-resolution molecular envelopes of DgrDps WT and ∆N variants for each condition tested. A restriction of P23 symmetry was set to reflect the typical Dps dodecamer quaternary structure. The models that better fit the experimental data (GASBOR models for DgrDps WT and DAMMIN models for DgrDps ∆N) are shown in Figure 4D, superimposed with the three-dimensional homology model for DgrDps, modelled using *D. radiodurans* Dps1 (PDB: 2C2U) as a template, which was docked into each envelope. The end-to-end diameter of each model can be found in Table 2. All models exhibit a spherical core of ~10 nm wide representing the dodecamer cage, together with a flexible shape at the outer regions of the molecule. The molecular envelope of the WT protein at the lowest ionic strength condition tested (50 mM NaCl, red model in Figure 4D) is representative of a “closed” tail conformation, pressed against the dodecamer cage with an end-to-end diameter of 14.1 nm and higher occupancy near the cage. As the ionic strength increases (from 50 mM NaCl to 480 mM NaCl), the flexible region of the model starts extending outwards and tail occupancy gradually shifts from the areas closer to the cage into an “open” distended conformation, with twelve finger-like protrusions and an end-to-end diameter of 18.0 nm. This outward facing conformation, which is more exposed to the solvent, is likely stabilized by the electrostatic interactions between the charged residues present at the distal end of the N-terminal tail and the ions in the buffer. Due to the lack of N-terminal tails, the molecular model of the DgrDps ∆N variant can be described as a spherical envelope with a diameter of 9.0 nm, regardless of the ionic strength of the buffer (50 mM NaCl in light gray and 230 mM in dark gray), which suggests that the conformational dynamics observed for the WT protein are likely restricted to the N-terminal tails, with the dodecamer cage remaining largely unaffected.

Overall, the shape and tail conformation calculated for each ionic strength condition tested agree with the results previously obtained from the SEC and DLS experiments, supporting the correlation between the ionic strength of the buffer and the overall protein structure, and a gradual rearrangement of the N-terminal tails between a compact, inward facing conformation and an extended, solvent-accessible conformation, stabilized by the increasing number of electrostatic interactions.

### 2.3. Detection of a Novel Iron-Binding Site in the N-Terminal Tail

**Figure 5:**
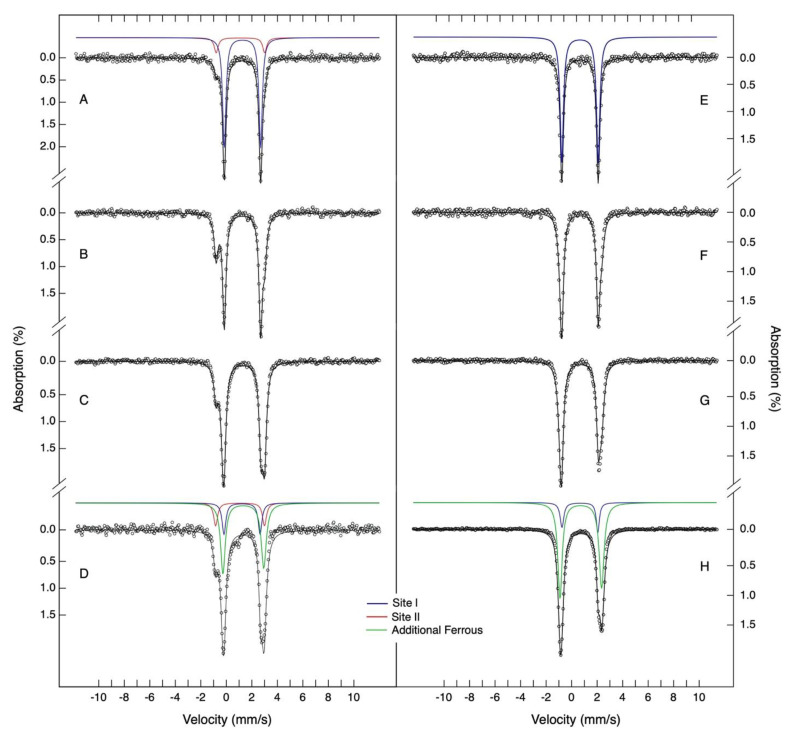
Mössbauer spectra of ferrous iron loading experiments for DgrDps WT and ∆N. Samples of proteins apo-form were incubated with different molar ratios of ferrous ^57^Fe (from 6 to 48 Fe per protein, WT in spectra (**A**–**D**) and ∆N in spectra (**E**–**H**) in anaerobic conditions for 20 min. Samples were prepared in buffers containing 200 mM MOPS pH 7.0 and 200 mM NaCl. The spectra were recorded at 80 K, with no external magnetic field applied. The solid lines are theoretical simulations using the parameters listed in Table 3. Site I, II, and additional ferrous species are presented in blue, red, and green, respectively.

The spectroscopic characterization and mechanistic understanding of the iron centers present in Dps proteins, a member of the ferritin family, is of uttermost interest. ^57^Fe Mössbauer spectroscopy is the technique of choice to probe the iron sites, since oxidation state, spin state and coordination environment can be inferred from spectral analysis. Moreover, no iron state is silent and a direct correspondence between the signal area relative percentage and iron species concentration can be established. Taking into consideration the putative presence of a metal-binding site in the proximal end of the N-terminal tail of DgrDps, both WT and ∆N variant were titrated with ^57^Fe^2+^ ions (from 6 to 48 Fe per protein) inside an anaerobic chamber for spectroscopic characterization of their iron-binding sites, using Mössbauer spectroscopy (Figure 5).

The Mössbauer spectrum of DgrDps WT, obtained for the lowest iron per protein ratio (6 Fe/DgrDps, see spectrum of Figure 5A), is well described by two quadrupole doublets, with parameters indicative of high-spin ferrous compounds. The major quadrupole doublet, with parameters of *δ* equal to 1.27 ± 0.02 mm/s and Δ*E*_Q_ equal to 2.85 ± 0.03 mm/s, has been already identified in a previous work for the Dps from *Marinobacter hydrocarbonoclasticus* [33]. These iron site parameters (henceforth designated by site I) are in accordance with ferrous centers with nitrogen/oxygen coordination, being similar to the ones observed for the ferrous state of the protocatechuate 4,5-dioxygenase active site [34]. In this enzyme, the iron site has a ligand geometry, approximately trigonal bipyramidal, with two nitrogen and three oxygen atoms in the first sphere of coordination [35]. This is not far from the proposed coordination of iron in Dps ferroxidase centers. An additional quadrupole doublet, with a distinct low-energy absorption line at −0.80 mm/s, is also detectable. A least squares fit of the data, assuming two quadrupole doublets, yields an iron site with *δ* = 1.10 ± 0.02 mm/s and ∆*E*_Q_ = 3.82 ± 0.02 mm/s (from now on designated by site II). These parameters are less common for non-heme iron proteins. Indeed, the low value obtained for the isomer shift is usually indicative of a mixed coordination environment with O/N and S ligands [36]. This is, however, impossible in the case of DgrDps, since no cysteinyl residues exist. A possible example of site II coordination is the one observed for the soybean lipoxygenase ferrous active site, which has Mössbauer parameters similar to the ones observed for site II [37]. In this case, the iron site has the following five ligands: three nitrogen atoms from histidine side chains, one oxygen atom from the N-terminal carboxylate group and one water molecule. The less rigid, asymmetric coordination sphere is well attuned with the putative tail-binding site. As an attempt to ascertain this possibility, the Mössbauer spectrum of a ∆N variant sample, which reacted with six iron ions per protein ratio, was acquired in the same experimental conditions (Figure 5E). The spectrum shows a single doublet that, within experimental uncertainties, is identical to the one obtained for site I. This, plus the fact that site II is absent, points to the conclusion that site II ligands reside in the N-terminal tail region of the protein.

**Figure 6:**
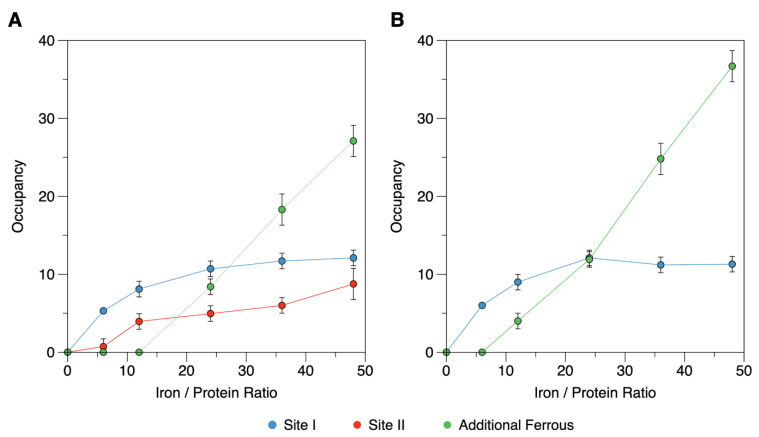
Occupancy of each type of ferrous iron in the iron-loaded Mössbauer samples of DgrDps WT (**A**) and ∆N variant (**B**). The deconvolution of each spectrum into three different ferrous species allows the determination of the area percentage of each signal, which in turn can be used to calculate the stoichiometry of each type of ferrous signal per protein, considering the total amount of Fe per protein added. Site I, II, and additional ferrous species are in blue, red, and green, respectively.

To further characterize the ferrous-binding sites of DgrDps, protein samples loaded with 12, 24, 36 and 48 Fe^2+^ ions per protein were prepared in anaerobic conditions. In the case of DgrDps WT (Figure 5B,C), it is noticeable that further addition of ferrous ions will first occupy site I and also site II, although with lower apparent affinity under the conditions of this study (Figure 6A, blue and red lines). For the 24 and 48 Fe^2+^ ions per protein ratios, an additional broad quadrupole doublet appears. A reasonable agreement with the experimental data is obtained using least squares fit analysis with three quadrupole doublets. The additional quadrupole doublet parameters are characteristic of high-spin ferrous ions (*δ* = 1.35 ± 0.02 mm/s; ∆*E*_Q_ = 3.23 ± 0.03 mm/s). While it is not possible to know if all of these additional iron ions are chelated by the protein, the Mössbauer parameters point to the existence of differences in the coordination environments and local structures of this ferrous species. It is, thus, appropriate to think that the additional ferrous species is explained by non-specific ferrous binding in the protein channels and/or simply solvated ions. In this set of experiments, the occupancy of site I maximizes at an apparent stoichiometry of ~12 Fe/protein, while site II reaches ~9 Fe/protein.

A similar analysis was carried out for the equivalent samples of the ∆N variant, in which case only two quadrupole doublets corresponding to site I and additional ferrous species were detected (Figure 5F–H). Noticeably, the site II subspectrum was absent for all the samples, while site I maximized at 11 Fe/protein (Figure 6B).

A comprehensive summary of the parameters and percentages of each species can be found in Table 3. Taken together, the occupancy values determined for the WT and ∆N variant datasets are consistent with the binding sites in the ferroxidase centers (site I) and N-terminal tail (site II) that is occupied independently and saturates at a stoichiometry close to 12 Fe each.

## 3. Discussion

Since their discovery, Dps proteins have been directly linked to the prokaryotic defense mechanism against starvation, oxidative stress, and DNA damage through their bifunctional property of DNA binding and iron biomineralization. The role of the N- and C-terminal tails in the oligomerization and DNA binding properties of these proteins is still a relatively new, but evidently important, research topic. Recent works in the literature have focused on the significance of the length and residue composition of the N-terminal tail from different Dps homologues, as well as their isoelectric point and divalent metal interactions [3,10,11,16,17,22]. In this work, we present a multi-technical approach to further understand the impact of ionic strength on the conformation of the N-terminal tails of *D. grandis* Dps, which possess one of the lengthiest N-terminal extensions within the mini-ferritin genomic databases.

The results presented herein have shown that changes in the ionic strength, ranging from 50 to ~500 mM progressively, alter the conformation of the N-terminal tails from a compact conformation (~14 nm wide), with tails pressed against the dodecamer cage, into an extended conformation (~18 nm wide), with increased hydrodynamic radius and solvent accessibility, all without impacting the tertiary structure and assembly of the dodecameric protein core shell. The conformations and molecular models calculated from our SAXS data not only agree with the results from the SEC and DLS experiments, but are also consistent with the models obtained for the extended and truncated conformations of *D. radiodurans* Dps1 and Dps2 [23] and the more compact conformation found for *E. coli* Dps, in the presence of Mg^2+^ and Fe^2+^ ions [24,25]. Buffer composition has also been previously described as key to the ability of Dps and DNA to form co-crystals and achieve low- and high-order condensation, with higher levels of NaCl, Mg^2+^ or Fe^2+^ ions in the solvent, disrupting DNA binding and abolishing co-crystallization [38,39,40]. Taken together, these observations suggest that the DNA binding and condensation properties of Dps proteins are related to the conformation of the N- and possibly C-terminal tails and their electrostatic interactions with the solvent, with buffer ionic strength being directly responsible for promoting different tail conformations and, subsequently, influencing the DNA-binding properties of the protein. Ultimately, this conclusion prompts the search for other modulators of Dps tail dynamics, which given the complexity of the bacterial cytosol and nucleoid composition, are likely not limited to the salt or divalent ions concentration. The inherent length and solvent accessibility of these extensions may also promote the interaction with other biomolecules involved in DNA shaping and protection.

Furthermore, the detection of a novel tail specific high-spin ferrous iron site, by means of Mössbauer spectroscopy, prompts the investigation of the metal-binding properties of the N-terminal tail of *D. grandis* Dps, which conceivably involves a metal-binding site, similar to the one found in the proximal end of the N-terminal tail in the crystal structure of *D. radiodurans* Dps1. Based on a comparative analysis of the amino acid sequences of these proteins, the residues that compose the metal-binding site found in the N-terminal tail of *D. radiodurans* Dps1 [Asp36-(X)_2_-His39-(X)_10_-His50-(X)_4_-Glu55], are conserved in DgrDps and correspond to Asp34, His37, His48 and Glu53, respectively. The functional role of this metal site is still unknown.

Further research is needed to fully characterize the structural dynamics of the N-terminal tails of Dps systems and to understand why there is such a high degree of evolutionary diversity in the primary sequence length and composition of these extensions, given their influence on the activity of mini-ferritins.

## 4. Materials and Methods

### 4.1. Gene Cloning, Protein Production and Purification

A cloning vector containing the ORF from gene DEIGR_102924 (Uniprot) of the genome (GenBank BCMS01000001.1) of *Deinococcus grandis* ATCC:43672 (NCBI: txid57498) was obtained via Invitrogen GeneArt Gene Synthesis services (Thermo Fischer Scientific, Waltham, MA, USA). The DNA fragment was transferred to a pET-21c(+) overexpression vector (Novagen, MERCK, Darmstadt, Germany) using the NdeI and NotI restriction sites with a stop codon before the 3′ restriction site, coding for the full wild-type DgrDps (NCBI Reference Sequence of WP_058978256.1). The same process was applied for the construction of the expression vector for the tailless variant ∆N, lacking the first 46 amino acid residues, designed by the inference from the three-dimensional homology model of the protein [28,41].

The recombinant expression of DgrDps WT was achieved by transforming competent *E. coli* BL21(DE3) cells with the pET21c:DgrDpsWT vector. The transformants were grown in an LB medium (25 g/L, NZYTech, Lisbon, Portugal) containing 100 µg/mL of ampicillin in an orbital shaker, set at 37 °C, 190 rpm until the optical density at 600 nm reached ~0.7. At that point, gene expression was induced with 0.5 mM of IPTG (isopropyl β-d-1-thiogalactopyranoside) for 3 h at 37 °C. The cells were harvested by centrifugation and resuspended in a buffer containing 50 mM Tris-HCl pH 7.5, 150 mM NaCl and 1 mM EDTA. The cell extract was obtained after 6 cycles of sonication on ice (Labsonic M, Sartorius, Goettingen, Germany). Protease inhibitors (1 mM benzamidine and 1 mM PMSF) were added to the extract, which was then centrifuged at 15,000× *g* for 30 min (Z36HK, HERMLE, Labortechnik, Wehingen, Germany Z36HK). Protein production and cell fractionation were evaluated by SDS-PAGE. Since DgrDps WT was produced in inclusion bodies, the pellet was resuspended in 10 volumes of buffer containing 50 mM Tris-HCl pH 7.5, 250 mM NaCl, 1% Triton X-100 and 1 M urea. The suspension was incubated for 1 h at room temperature with gentle rocking, before a second centrifugation step at 15,000× *g* for 30 min to remove the non-soluble particles. The supernatant was then dialyzed overnight at 4 °C against a buffer containing 10 mM Tris-HCl pH 7.5 and 1 M urea for purification. Contrarily, DgrDps ∆N was produced in the soluble form. Thus, the supernatant of the first centrifugation step after cell lysis was collected and subsequently dialyzed against 10 mM Tris-HCl pH 7.5, overnight at 4 °C.

From this point forward, protein purification of both the DgrDps WT and ∆N variant followed the same protocol. All chromatographic steps described below were performed on an ÄKTA Prime Plus system (Cytiva, Marlborough, MA, USA). First, the dialyzed fraction was loaded into a DEAE-Sepharose Fast Flow column (2.6 × 30 cm resin bed, XK-26/40, Cytiva), pre-equilibrated with 10 mM Tris-HCl pH 7.5 (buffer A). After elution of the flow-through fraction, the adsorbed proteins were eluted with a linear NaCl gradient between 0 and 500 mM with buffer B (10 mM Tris-HCl pH 7.5 and 500 mM NaCl). The elution profile following the absorbance at 280 nm was assessed by SDS-PAGE. The fractions containing the DgrDps proteins (WT or ∆N variant) were pooled, concentrated using Vivacell or Vivaspin concentrators (Sartorius) and dialyzed overnight against 10 mM Tris-HCl pH 7.5. At this point, the protein fraction was loaded into a Resource Q 6 mL column (Cytiva) for a second high resolution ionic exchange step. The elution was performed as above. Pure DgrDps fractions were pooled, concentrated, and ultimately injected into a Superdex 200 prep grade size-exclusion chromatography (SEC) column (1.6 × 60 cm resin bed, XK-16/60, Cytiva) for buffer exchange and final oligomer state and homogeneity assessment. The protein was eluted with 200 mM MOPS pH 7.0 and 200 mM NaCl buffer. The pure fractions were concentrated and stored at –80 °C until further use.

As mentioned, all steps were analyzed by SDS-PAGE, performed using hand cast 12.5% polyacrylamide gels. Low Molecular Weight Protein Marker II or NZYColour Protein Marker I (NZYTech, Lisbon, Portugal) were used as markers and BlueSafe (NZYTech) as protein stains. The protein concentration of the DgrDps WT and ∆N stock solutions and samples was determined using the theoretical molar extinction coefficient at 280 nm of the protein monomer, which is ε_280nm_ = 21,430 M^−1^ cm^−1^ for both constructs, according to ExPASy’s ProtParam (Swiss Institute of Bioinformatics, Lausanne, Switzerland).

### 4.2. Analytical Size-Exclusion Chromatography

A pre-packed high-performance Superdex 200 10/300 GL column (Cytiva) was calibrated using a set of gel filtration protein markers (NZYTech), containing arabinoxylase 5A (92.9 kDa), bovine serum albumin (66.4 kDa), ovalbumin (44.3 kDa), carbonic anhydrase (29.4 kDa) and ribonuclease A (13.7 kDa), together with ferritin from horse spleen (529 kDa) and catalase (232 kDa). The elution of each standard was performed at a flow rate of 0.5 mL/min, with a 200 mM MOPS pH 7.0 and 200 mM NaCl buffer. The void volume of the column was determined for each separate standard injection by mixing ot with <1 mg of blue dextran 2000 (NZYTech). The elution profiles of the standard set were used to determine the apparent molecular mass and the hydrodynamic radius (Stokes radius, *R_S_*) of the protein calibration curves, as demonstrated by Equations (1) and (2) [42], which are as follows:

(1) $$Log_{10}\left(MM\right)=-1.546\frac{V_{e}}{V_{o}}+7.677$$

(2) $$Log_{10}\left(R_{S}\right)=-1.028\;\frac{V_{e}-V_{o}}{V_{t}-V_{o}}+0.99$$

where *MM* is the apparent molecular mass in kDa, *R_S_* is the Stokes radius in nm, *V_e_* is the elution volume of the protein, *V_o_* is the void volume and *V_t_* is the total volume of the packed bed column.

The quaternary structure, apparent molecular mass and apparent Stokes radius of DgrDps WT and ∆N variant samples at different ionic strength conditions were estimated by preparing samples with approximately 1 mg of protein, dialyzing them against the appropriate buffer, clearing the sample by centrifugation at 15,000× *g* for 5 min, adding blue dextran to the resulting supernatant and injecting the samples into the Superdex 200 10/300 GL column pre-equilibrated with the same buffer, at a flow rate of 0.5 mL/min.

### 4.3. Dynamic Light Scattering

The hydrodynamic diameter (Z-average) and polydispersity index (PI) of 1 mg/mL protein samples in a 50 mM MOPS pH 7.0 buffer, containing variable concentrations of NaCl, were assessed by dynamic light scattering (DLS). The samples were centrifuged for 30 min at 14,000× *g* at room temperature, before measurements in a HORIBA SZ-100 nanoparticle analyzer (HORIBA, Kyoto, Japan), equipped with a 10 mW 532 nm laser and detection at a scattering angle of 90° during 2 min at 25 °C, in at least triplicates. Data were analyzed using the equipment built-in software assuming a standard monodisperse form of distribution, using a particle refractive index of 1.6 (organic sample) and water settings as dispersion medium (refractive index of 1.333).

### 4.4. Circular Dichroism Spectroscopy

Synchrotron radiation circular dichroism (SRCD) spectra of DgrDps WT samples were acquired at the AU-CD beamline at the ASTRID2 synchrotron radiation facility at ISA, the Department of Physics and Astronomy, Aarhus University, Denmark. The spectra of 1 mg/mL protein samples, freshly dialyzed against the buffer of interest (10 mM MOPS pH 7.0, containing either 60 mM or 240 mM of NaF), were recorded in 1 nm steps and a dwell time of 2.1 s per step, in triplicates, using a nominally 0.01 cm pathlength quartz cell (SUPRASIL, Hellma GmbH, Müllheim, Germany) for the wavelength range of 170–280 nm, at 25 °C. The actual pathlength of the cell was determined to be 0.01008 cm by an interference technique [43]. The molar circular dichroism, ∆ε, for each spectrum was calculated using the protein concentration estimated from the absorbance at 205 nm and the protein molar extinction coefficient at the same wavelength [44]. The secondary structure contents of the proteins in the different buffer conditions were determined using DichroWeb, an online CD structure analysis tool [45], with CDSSTR as the analysis program and SP175 as the reference set.

### 4.5. Iron Oxidation Activity Assays

The iron oxidation (ferroxidation and mineralization) activity of recombinant DgrDps WT and DgrDps ∆N proteins was assessed by monitoring the absorbance at 340 nm (Evolution 300 UV–Vis spectrophotometer, Thermo Fischer Scientific, Waltham, MA, USA), after adding different iron substrate concentrations to protein samples in oxygen saturation conditions. The samples containing 0.3 µM of DgrDps (dodecamer) in 200 mM MOPS pH 7.0 and 200 mM NaCl were transferred to a 1 cm quartz cuvette (SUPRASIL, Hellma GmbH, Müllheim, Germany) for baseline measurement, followed by a single addition of different iron/protein amounts (from 48 to 480) of a ferrous sulphate solution, prepared in acidic Milli-Q water at pH 3.0 and quantified by the 1,10-phenantroline method (*ε*_510nm_ = 11,100 M^−1^cm^−1^) [46].

### 4.6. Small-Angle X-ray Scattering Data Acquisition and Analysis

Synchrotron SAXS data was measured at beamline P12, operated by EMBL Hamburg at the PETRA III storage ring [30] (DESY, Hamburg, Germany). Scattered X-ray photons (λ = 0.124 nm) were collected on a Pilatus 6M detector (DECTRIS, Baden, Switzerland), with a sample to detector distance of 3 m. The scattering intensity I(s) was recorded for 0.02 < s < 7.0 nm^−1^, with s = (4πsin2θ)/λ.

For each NaCl concentration, DgrDps WT and ∆N were measured at different protein concentrations (1, 2, 5 and 10 mg/mL), together with their corresponding buffers. Data were collected using the robotic sample changer [47]. In addition, DgrDps WT and ∆N protein samples at ~10 mg/mL initial concentration were measured by SEC-SAXS, using a Superdex 200 Increase 5/150 GL column (Cytiva) [48] in 50 mM MOPS pH 7.0 containing varying concentrations of NaCl, between 50 and 480 mM. For the sample changer measurements, 40 frames of 100 ms were collected. Data were reduced using the SASflow pipeline [49]. The 2D images were radially averaged and frames with no trace of radiation damage were averaged and used for further processing. For each protein sample, the data collected on the corresponding buffer was subtracted and curves were scaled to the protein concentration. For the SEC-SAXS measurements, 3000 successive 1 s frames were collected while the sample eluted through the column. CHROMIXS [50] was used to visualize the SEC-SAXS data and select the relevant sample and buffer frames.

The one-dimensional data was analyzed and treated using the ATSAS program suite [51] (PRIMUS, CHROMIX, GNOM) for the analysis of the scattering profiles, Guinier plots, Kratky plots and pair distance distribution (*P*(*r*)) calculations. The ab initio models of the DgrDps WT and ∆N variant were obtained from the SAXS data using GASBOR [52] and DAMMIN [53]. At least 10 iterations of both programs were independently performed to validate the models. A representative of the most typical model obtained was used in the figures. The three-dimensional envelope maps for each sample were computed, docked and represented using ChimeraX [54].

### 4.7. Mössbauer Spectroscopy

Mössbauer samples of the DgrDps WT and ∆N variant were prepared inside an anaerobic glovebox (<4 ppm O_2_; MBLab, MBraun, Garchig, Germany). The protein samples, containing 166 µM of either DgrDps WT or ∆N (dodecamer) in 200 mM MOPS pH 7.0 buffer with 200 mM NaCl, were incubated with ferrous sulphate at a molar ratio of 6, 12, 24, 36 or 48 Fe/Dps (1 to 8 mM Fe) for 20 min at room temperature, before being frozen in liquid nitrogen. The ^57^FeSO_4_ solution was prepared by the acidic dissolution of a ^57^Fe metal foil (>95% enrichment) with H_2_SO_4_ [55], followed by iron quantification by complexation with 1,10-phenantroline, as described before [46].

Mössbauer spectra were acquired at 80 K in the absence of an external magnetic field in transmission mode, using a conventional constant acceleration spectrometer and a 25- mCi ^57^ Co source in a Rh matrix. The velocity scale was calibrated using a α-Fe foil at room temperature and the isomer shift values (*δ*) were given relative to this standard. An analysis was carried out using the WMOSS software.

## Figures and Tables

**Table 1:** Macromolecular structure characteristics of DgrDps WT when varying the ionic strength of the buffer, as per SEC, DLS, and SRCD analysis.

	**[NaCl] (mM)**	**V_e_ (mL)**	**Apparent R_S_ (nm)**	**Apparent MM (kDa)**
**SEC**	50	12.2	4.9	222
	80	11.9	5.3	290
	150	11.1	5.9	359
	230	10.8	6.2	405
	480	10.5	6.4	446
	**[NaCl] (mM)**	**Z-Average (nm)**	**SD**	**PI**
**DLS**	50	12.9	0.63	0.205
	80	13.1	0.51	0.312
	150	14.4	0.62	0.187
	230	16.3	0.63	0.275
	480	16.5	0.92	0.281
	**[NaF] (mM)**	**α-Helix (%)**	**β-Sheet (%)**	**Unordered (%)**
**SRCD**	60	64	5	31
	240	63	5	32

V_e_—elution volume; R_S_—Stokes radius; Z-average—intensity weighted hydrodynamic size; SD—standard deviation; MM—molecular mass; PI—polydispersity index.

**Table 2:** Data collection and SAXS parameters for DgrDps WT and ∆N variants.

	WT				∆N	
[NaCl]	50	80	230	480	50	230
**Data Collection**						
Beamline	P12, PETRAIII					
Beam dimensions (mm)	0.2 × 0.05					
Detector	Pilatus 6M					
Wavelength (Å)	1.24					
*q* range (nm^−1^)	0.02–7.0					
Concentration (mg/mL, batch)				1–10		
Concentration (mg/mL, SEC)	8	9	10		10	10
Exposure time per frame (s)	1	1	1	0.1	1	1
**Structural Parameters**						
I(0) from Guinier	11,800	20,311	74,700	0.19	50,671	46,710
*R_g_* from Guinier (nm)	4.38	4.47	4.45	4.67	3.75	3.75
I(0) from *P*(*r*)	11,840	20,310	75,070	0.17	50,870	46,840
*R_g_* from *P*(*r*)	4.43	4.52	4.54	4.87	3.72	3.72
*D*_max_ from *P*(*r*)	16.09	17.10	20.63	20.46	9.10	8.80
Porod volume (AutoRG) (nm^3^)	335.9	436.7	427.3	418.4	290.7	296.6
Porod volume (GNOM) (nm^3^)	418.5	475.0	438.2	430.3	288.0	291.2
**Molecular Mass**						
Estimation from data (kDa)	208.0	242.0	318.5	318.5	208.0	208.0
Theoretical dodecamer (kDa)	270.4	-	-	-	216.5	-
**Model χ^2^**						
DAMMIN	1.99	3.33	2.09	2.65	2.86	6.66
GASBOR	3.63	4.91	4.32	3.34	97.49	47.02
Model diameter (nm)	14.05	17.1	17.5	18.0	9.0	8.8
**Software**						
1D data processing	PRIMUS					
*P*(*r*) analysis	GNOM					
*Ab initio* methods	GASBOR				DAMMIN	
3D model visualization	ChimeraX					

**Table 3:** Mössbauer parameters of high-spin ferrous species detected in loading experiments using DgrDps WT and ∆N. Contribution of each species in each spectrum after deconvolution is given in percentage and occupancy values. Values in brackets are uncertainties of the last significant digits.

**Spectroscopic Parameters for the Ferrous Species in Iron Loaded DgrDps WT and ∆N Variant**							
		**Site I**		**Site II**		**Additional Ferrous**	
*δ* (mm/s)		1.27 (2)		1.10 (2)		1.35 (2)	
∆*E*_Q_ (mm/s)		2.85 (3)		3.82 (2)		3.23 (3)	
Linewidth (mm/s)		0.32 (2)		0.37 (2)		0.42 (4)	
**Percentages of the Iron Absorption of the Ferrous Species**							
	**Iron Ratio**	**%**	**Occupancy**	**%**	**Occupancy**	**%**	**Occupancy**
WT	6	88 (2)	5 (0)	12 (2)	1 (1)		
	12	67 (2)	8 (1)	33 (2)	4 (1)		
	24	44 (2)	10 (1)	21 (2)	5 (1)	35 (5)	8 (1)
	36	32 (2)	12 (1)	17 (2)	6 (1)	51 (5)	18 (2)
	48	25 (2)	12 (1)	19 (2)	9 (2)	56 (5)	27 (2)
∆N	6	100	6 (0)				
	12	68 (2)	8 (1)			32 (5)	4 (1)
	24	50 (2)	12 (1)			50 (5)	12 (1)
	36	31(2)	11 (1)			69 (5)	25 (2)
	48	23 (2)	11 (1)			76 (5)	37 (2)

## Data Availability

The SAXS datasets and models were deposited in the Small Angle Scattering Biological Data Bank (SASBDB), under the accession numbers SASDNY7, SASDNZ7, SASDN28, SASDN38, SASDN48 and SASDN58.

## Supplementary Materials

The following supporting information can be downloaded at: https://www.mdpi.com/article/10.3390/ijms23094871/s1.
