# Supplementary material for: The Conformation of the N-Terminal Tails of Deinococcus grandis Dps Is Modulated by the Ionic Strength
Source: Int J Mol Sci. 2022 Apr 28;23(9):4871. doi: 10.3390/ijms23094871 (PMC9103930; doi:10.3390/ijms23094871)
Supplement: Supplementary file 1 [file ijms-23-04871-s001.zip › ijms-1689354-supplementary.pdf]

International Journal of Molecular Sciences

MDPI

Supplementary Material

The Conformation of the N-Terminal Tails of *Deinococcus grandis* Dps Is Modulated by the Ionic Strength

João P. L. Guerra [1,2], Clement E. Blanchet [3], Bruno J. C. Vieira [4], Ana V. Almeida [1,2], João C. Waerenborgh [4], Nykola C. Jones [5], Søren V. Hoffmann [5], Pedro Tavares [1,2,*] and Alice S. Pereira [1,2,*]

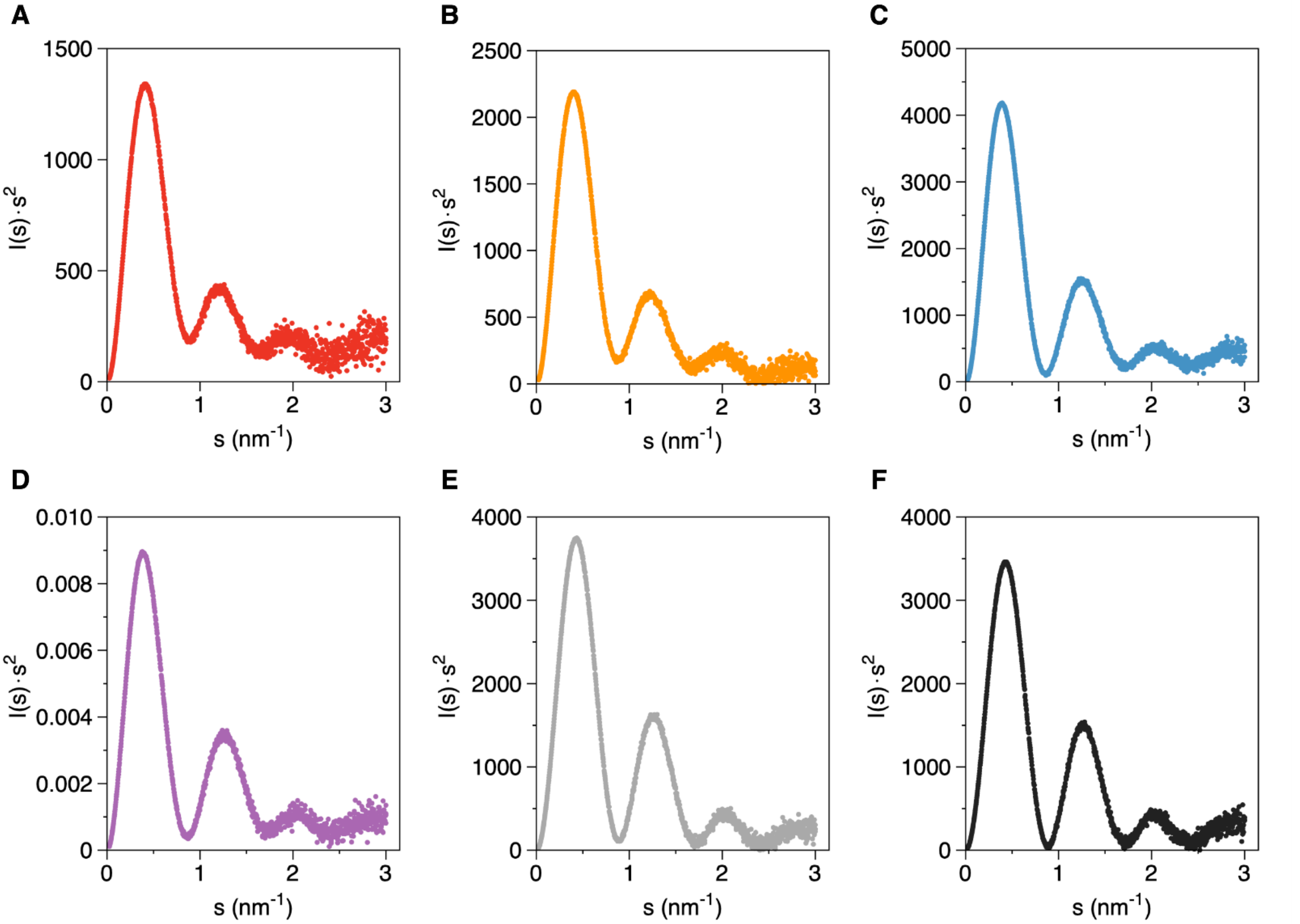

Figure S1. Kratky plots generated from the experimental scattering curves of DgrDps samples in 50 mM MOPS pH 7.0 buffer containing varying concentrations of NaCl: 50 mM (A), 80 mM (B), 230 mM (C) and 480 mM (D) for DgrDps WT and, for the ΔN protein variant, either 50 mM (E) or 230 mM NaCl (F).
